# Markers of positive affect and brain state synchrony discriminate melancholic from non-melancholic depression using naturalistic stimuli

**DOI:** 10.1038/s41380-024-02699-y

**Published:** 2024-08-27

**Authors:** Philip E. Mosley, Johan N. van der Meer, Lachlan H. W. Hamilton, Jurgen Fripp, Stephen Parker, Jayson Jeganathan, Michael Breakspear, Richard Parker, Rebecca Holland, Brittany L. Mitchell, Enda Byrne, Ian B. Hickie, Sarah E. Medland, Nicholas G. Martin, Luca Cocchi

**Affiliations:** 1 QIMR Berghofer Medical Research Institute, Herston, QLD Australia; 2 Queensland Brain Institute, University of Queensland, St Lucia, QLD Australia; 3 Australian eHealth Research Centre, CSIRO Health and Biosecurity, Herston, QLD Australia; 4 Faculty of Medicine, School of Biomedical Sciences, University of Queensland, St Lucia, QLD Australia; 5 School of Information Systems, Queensland University of Technology, Kelvin Grove, QLD Australia; 6 Metro North Mental Health, Royal Brisbane & Women’s Hospital, Herston, QLD Australia; 7 School of Psychology, College of Engineering, Science and the Environment, University of Newcastle, Newcastle, NSW Australia; 8 Brain Neuromodulation Research Program, Hunter Medical Research Institute, Newcastle, NSW Australia; 9 School of Medicine and Public Health, College of Medicine, Health and Wellbeing, University of Newcastle, Newcastle, NSW Australia; 10 Child Health Research Centre, University of Queensland, South Brisbane, QLD Australia; 11 Brain and Mind Centre, University of Sydney, Camperdown, NSW Australia; 12 School of Psychology, University of Queensland, St Lucia, QLD Australia; 13 School of Psychology and Counselling, Queensland University of Technology, Kelvin Grove, QLD Australia

**Keywords:** Neuroscience, Depression

## Abstract

Melancholia has been proposed as a qualitatively distinct depressive subtype associated with a characteristic symptom profile (psychomotor retardation, profound anhedonia) and a better response to biological therapies. Existing work has suggested that individuals with melancholia are blunted in their display of positive emotions and differ in their neural response to emotionally evocative stimuli. Here, we unify these brain and behavioural findings amongst a carefully phenotyped group of seventy depressed participants, drawn from an established Australian database (the Australian Genetics of Depression Study) and further enriched for melancholia (high ratings of psychomotor retardation and anhedonia). Melancholic (*n* = 30) or non-melancholic status (*n* = 40) was defined using a semi-structured interview (the Sydney Melancholia Prototype Index). Complex facial expressions were captured whilst participants watched a movie clip of a comedian and classified using a machine learning algorithm. Subsequently, the dynamics of sequential changes in brain activity were modelled during the viewing of an emotionally evocative movie in the MRI scanner. We found a quantitative reduction in positive facial expressivity amongst participants with melancholia, combined with differences in the synchronous expression of brain states during positive epochs of the movie. In non-melancholic depression, the display of positive affect was inversely related to the activity of cerebellar regions implicated in the processing of affect. However, this relationship was reduced in those with a melancholic phenotype. Our multimodal findings show differences in evaluative and motoric domains between melancholic and non-melancholic depression through engagement in ecologically valid tasks that evoke positive emotion. These findings provide new markers to stratify depression and an opportunity to support the development of targeted interventions.

## Introduction

A deeper phenotypic characterisation of persons with depression is needed, given that depression is one of the most common mental illnesses and a leading cause of disability and suicide [1]. Information that can guide the selection of appropriate therapies for individuals with depression is especially valuable because one-third of people still have clinically significant symptoms despite optimal drug therapy [2]. Melancholia has been proposed as a categorically distinct subtype of depression, marked by a characteristic phenotypic profile including pronounced psychomotor changes, profound anhedonia and cognitive slowing [3]. For persons with melancholia, depressed mood tends to arise ‘*out of the blue*’ rather than in response to antecedent psychosocial stressors. Moreover, if there are relevant life events, the depressive response is far greater than expected. Compared to non-melancholic depression, melancholic depression responds more robustly to antidepressant medication and neurostimulation as compared to psychotherapy (and with a lower likelihood of placebo response) [4]. Identification of melancholic features may thus facilitate the selection of the most effective therapy. However, existing classification systems position melancholia as a dimensional expression of severe depression, reducing its utility as a construct. This negatively affects the accuracy of subtyping and constrains the capacity for a diagnosis of melancholic depression to give reliable and robust predictions about treatment outcomes by modality (biological versus psychological) [5]. In part, this may reflect the lack of specificity in classification and the likely subsequent pooling of melancholic and non-melancholic subtypes in recent studies [6].

The Sydney Melancholia Prototype Index (SMPI) operationalises key motor, cognitive and illness correlates of melancholic depression in a format that can be delivered as part of a psychiatric assessment. Thus, the tool assists a clinician in discriminating whether a given person possesses the cross-sectional and longitudinal characteristics of melancholia [7]. Compared to a classification applied by a psychiatrist with full access to the clinical history and treatment records, the SMPI shows high sensitivity and specificity [8] and classifies fewer persons as melancholic in comparison to DSM symptom specifiers [9].

Given the proposed biological weighting of melancholia, there has been interest in pursuing a neural signature of this depressive subtype. Prior work has identified that the dynamics of brain network activity may be uniquely perturbed amongst participants with melancholia in a state of rest [10], when shifting from interoceptive to exteroceptive states [11], and when viewing emotionally evocative movie clips [12, 13]. While these studies provide support for the hypothesis that melancholia is a biologically distinct subtype of depression, these neural signatures have not yet been integrated with other dynamic markers.

Movie clips are naturalistic stimuli that probe the physiology of affective states in an ecologically valid manner. Thus, these stimuli are more sensitive to detecting group differences in the activity of behaviourally relevant brain networks [14, 15]. Recent work has shown that perceptual immersion in an engaging movie can reshape functional brain state transitions that recapitulate the content of movie scenes, are consistent over time, associate with distinct physiological changes, and correlate with a subjective appraisal of the movie [16]. In addition to these discriminatory brain states, psychophysical measures of dynamic changes in affect can also be used to discriminate melancholia in a naturalistic setting. The Facial Action Coding System (FACS) rates the activity of anatomical facial muscle groups (Action Units) on a quantitative scale, with emotional states associated with the co-occurrence of specific action units (e.g., opening the mouth and raising the cheeks to mediate a smile) [17]. Machine learning algorithms incorporating feature extraction can transform videos of faces into action unit time series that capture rich temporal dynamics underpinning the facial expression of emotions. Recent work has identified that, relative to healthy controls, people with melancholia exhibit impaired affective reactivity to positively (but not negatively) valenced clips [18].

Here, we extend prior work characterising brain and behavioural differences amongst a relatively large cohort of tightly classified depressed participants incorporating melancholic and non-melancholic presentations and enriched for a larger proportion of melancholic participants. Given the central role of anhedonia in melancholia, we focus on positive affect and emotion. First, we confirm a widespread ‘*positive blunting*’ of facial expressivity in persons with melancholic depression in response to a movie clip of a stand-up comedian. Second, we acquire functional neuroimaging (fMRI) whilst participants watch a movie evoking a range of complex emotional states to assess the emergence, maintenance of, and transition between distinct brain states related to the appraisal of positive scenes. We define a set of brain regions supporting the experience of positive emotion and show that the synchronous expression of and dynamic shifts in brain states differ between melancholic and non-melancholic participants. Furthermore, we find that a cerebellar region implicated in emotional processing mediates differences in the facial expression of positive affect between melancholic and non-melancholic depressed participants.

## Materials and methods

### Recruitment

This study was conducted at the QIMR Berghofer Medical Research Institute, Brisbane, Australia. All procedures were conducted in accordance with the study protocol approved by the Human Research Ethics Committee of QIMR Berghofer. Written, informed consent was obtained from all participants.

Participants were identified from the Australian Genetics of Depression Study (www.geneticsofdepression.org.au). This cohort comprises over 16,000 Australians who have been treated for depression and who have provided information about their symptoms, their response to therapy and a saliva sample for genetic analysis [19–21]. For this investigation, participants were approached if they: (i) lived within a 100 km radius of the institute, (ii) endorsed ongoing symptoms of depression and (iii) answered ‘*yes*’ to the study question: ‘*were you talking or moving much more slowly than is normal for you?*’. The latter criterion was included to enrich the sample for psychomotor retardation, which is a key aspect of the melancholic presentation. We aimed to recruit approximately equal groups of melancholic and non-melancholic depressed participants. Additional inclusion criteria included a primary diagnosis of major depressive disorder (as opposed to bipolar disorder) and age between 18 and 65. Exclusion criteria included a history of a primary psychotic illness, alcohol or illicit drug dependence and major neurological co-morbidity (including cerebrovascular disease). Participants were telephone-screened by the lead investigator before attending QIMR Berghofer for assessment, which took place in person over a single day. Our sample size was informed by previous work demonstrating  the detection of reliable brain state and facial dynamics in groups of <30 [18].

### Study procedures

**Fig. 1:**
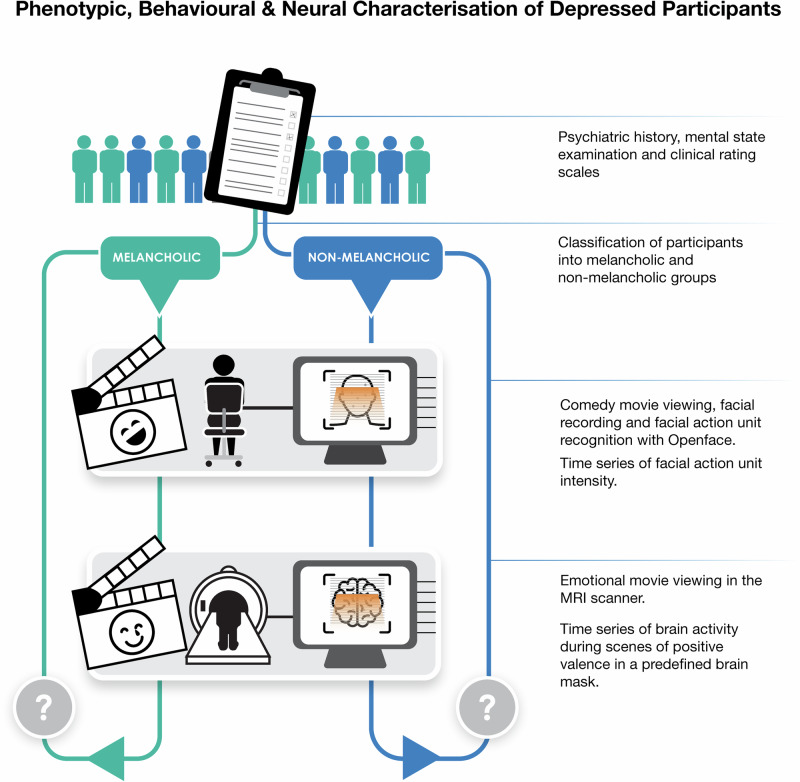
Study overview. Schematic of study procedures. All participants received the same testing procedures. Depressed participants were recruited from a national Australian database, and the sample was enriched for a higher likelihood of melancholia. Participants completed a clinical assessment with a psychiatrist, during which they were classified as melancholic or non-melancholic using a validated semi-structured interview (the Sydney Melancholia Prototype Index). Dynamic changes in positive facial expressivity were analysed using a convolutional neural network trained on the participant’s response to a movie clip of a stand-up comedian. We modelled brain activity in a set of brain regions associated with the positive evaluation of stimuli (see section ‘Materials and Methods’) during exposure to an emotionally evocative movie in the MRI scanner. Markers of facial affect and differences in patterns of brain activity were compared between melancholic and non-melancholic groups.

Figure 1 depicts an overview of the study. Seventy participants were recruited and assessed to confirm the diagnosis of depression according to DSM-5 criteria [22]. Depressive symptoms were rated using the Montgomery-Åsberg Depression Rating Scale (MADRS) [23, 24]. Anxiety symptoms were rated with the Hamilton Anxiety Inventory (HAM-A) [25]. Hedonic tone was rated with the Snaith-Hamilton Pleasure Scale (SHAPS) [26]. Participants with a melancholic subtype of depression were identified using the Sydney Melancholia Prototype Index (SMPI). This validated assessment instrument weights symptoms and signs of melancholia against non-melancholic depression [7, 27, 28]. Features suggestive of melancholia include loss of mood reactivity, psychomotor slowing, weight loss and a relative absence of precipitating stressors. We used a cut-off score of four or greater (as suggested by the authors of the scale) to classify participants as melancholic. Between-group differences were determined after accounting for multiple comparisons (false discovery rate approach, FDR) [29].

Due to the capacity for psychotropic medication to influence brain and facial dynamics, fluoxetine dose equivalences were calculated for antidepressant medication [30], and risperidone dose equivalences for antipsychotic medication [31].

Participants watched a 20-minute excerpt from a routine by a stand-up comedian (‘*Ricky Gervais: Animals*’) whilst their facial expressions were recorded using a video camera. This clip was selected because of its lack of dependence upon current affairs, its (relative) lack of profanity and its ability to act as a standalone excerpt without prior engagement with the comic. Further, we selected a humorous clip because of its sensitivity to detect a ‘*blunting*’ of positive affect, a key feature both of anhedonia and the psychomotor retardation defining melancholia, noted in the aforementioned experimental work as specifically perturbed in melancholia [18]. We decoded facial expressivity using the open-source machine learning algorithm OpenFace: (‘https://github.com/TadasBaltrusaitis/OpenFace/wiki*’*) [32]. This toolkit detects facial landmark location, head pose, eye gaze and activity within facial action units, providing a quantitative frame-by-frame readout of facial movement [33]. This allowed us to sensitively compare psychomotor activity between participants with melancholic and non-melancholic depression. The lead author supervised all recordings to ensure participant engagement in the movie. These clips are available through contacting the corresponding author.

At the Herston Imaging Research Facility, participants completed structural (MRI) and functional (fMRI) magnetic resonance imaging of the whole brain. fMRI data acquisition occurred whilst participants viewed a 20-minute emotionally evocative movie (‘*The Butterfly Circus*’) in the scanner. This short story depicts the journey of a man who is born without limbs but is encouraged by the showman of a renowned circus to discover his potential. The narrative architecture of The Butterfly Circus maps onto distinct acts of varying emotional tone, allowing the identification of brain states associated with processing scenes of positive valence. This movie is available through contacting the corresponding author.

### Facial movement analysis

The recording of each participant was sampled at 10 frames/s at a resolution of 1920 × 1200 using the inbuilt webcam on a Linux Ubuntu (20.04.6) machine. This relatively low frame rate is sufficient to capture relatively slow and persistent changes in facial expressivity during the appraisal of positive scenes, with the mean duration of micro-expressions in one study being three times this duration [34]. Using OpenFace, a time series for the dynamics of 16 facial action units (AUs) was extracted for each participant during the viewing of the comedy clip. OpenFace uses a convolutional neural network architecture to detect and track facial landmark points. After facial images were aligned to a common 112 × 112-pixel image, features from a histogram of oriented gradients were extracted and classified with a linear kernel support vector machine. Due to the potential for a meaningful reduction in facial expressivity amongst the depressed participants, we employed the -*au_static* flag. This prevented OpenFace from normalising each participant’s time series and consequently attenuating individual differences. AU 45 (‘*Blink*’) was extracted but not entered into the analysis pipeline as it is not directly related to the expression of emotions. The mean intensity of each AU across the sample was compared between melancholic and non-melancholic participants using a two-sample *t*-test corrected for false discovery rate (FDR) [29]. Confidence intervals were visualised on the time series with the 5th and 95th percentiles of 1000 bootstrap samples. We also calculated the first principal component of all 16 facial AUs and compared these between melancholic and non-melancholic participants. We isolated facial AUs exhibiting a statistically significant difference between groups and depicted these on an avatar using FACSvatar [35] and FACSHuman [36]. The avatar also presented the first principal component across all 16 AUs.

### Neuroimaging acquisition and preprocessing

We used a 3-Tesla Siemens Prisma MRI scanner with a 64-channel head coil to acquire whole-brain functional and structural (MRI) data. fMRI EPI data: multiband eight acceleration, repetition time (TR) 810 ms, echo time (TE) 30 ms, flip angle (FA) 53°, field of view (FOV) 212 × 212 mm, pixel bandwidth 2050 Hz, 72 axial slices and 2 mm^3^ voxel resolution. Spin-echo field maps in AP and PA directions were acquired with identical sequence parameters as the fMRI EPI data. MRI: T1-weighted MPRAGE structural image (TE 2.98 ms, TR 1900 ms, FA 9°, FOV 256 × 256 mm and voxel size 1 mm^3^).

We used fMRIPrep 22.0.2 [37], based on Nipype 1.8.5 [38], to preprocess the data. The pipeline corrects for intensity non-uniformity, skull-strips and segments the data into cerebrospinal fluid (CSF), white matter (WM) and grey matter (GM). Brain surfaces were reconstructed using FreeSurfer 7.2.0 [39] and spatial normalisation into standard space was performed with ANTS 2.3.3. Functional images were distortion corrected, slice-time corrected and co-registered to the structural image using boundary-based registration [40]. After spatial preprocessing, we used Nilearn version 0.5.0 to perform bandpass filtering (0.01–0.15 Hz), and regress the WM signal, CSF signal and head motion parameters. For the GLM analyses linking facial movement to brain activity, data were spatially smoothed (6 mm), and no low-pass temporal filtering was applied.

### Hidden Markov model and brain states supporting the processing of positive stimuli

We employed a Hidden Markov Model (HMM) to decompose dynamic fluctuations in fMRI signal into a sequence of discrete brain states that switch and recur over time according to a transition probability matrix [16].

Motivated by the link between melancholia and anhedonia, we were interested in studying brain states related to processing movie scenes of positive valence. Specifically, we tested the hypothesis that melancholic and non-melancholic subtypes differ in the synchronous expression of brain states related to the appraisal of positive scenes. Thus, our core analysis considered the expression of brain states in 22 movie periods with a positive tone and an uplifting narrative (see Supplementary Table 1). Brain states linked to the appraisal of positive scenes were inferred by extracting fMRI signals across the duration of the whole movie (ensuring that positive brain states were defined based on a broader repertoire of context-driven changes in fMRI activity) within pre-defined regions of interest (a positive valence map). The positive valance map was defined using the meta-analytic tool Neuroquery.org (querying the keywords ‘*feeling, happy, positive, satisfaction, valence, pleasure*’) [41]. As a result, 25 brain regions of interest (ROIs) were identified using Talairach Daemon [42] and the Melbourne subcortex atlas [43]. These regions included the amygdala, anterior cingulate cortex, insular cortex, fusiform gyrus, hippocampus, orbitofrontal cortex, thalamus, ventral striatum and right-hemispheric inferior frontal gyrus (full list in Supplementary Table 2 and Supplementary Fig. 1). For each participant, fMRI time series were obtained from all ROI by (i) calculating the mean signal within the ROI, (ii) removing time series associated with the first 10 volumes and last 35 volumes (time periods during which the movie was not played), (iii) demeaning the signal and (iv) scaling the resulting time series by its standard deviation.

Brain states were inferred using the HMM-MAR Matlab toolbox (https://github.com/OHBA-analysis/HMM-MAR; commit 721993a). The variational Bayesian inversion on concatenated and preprocessed ROI time series was completed using 500 training cycles. Specifically, time-by-region matrices were concatenated across participants to form a group-level input to the HMM. This strategy allowed us to compare the expression of similar brain states across the melancholic and non-melancholic groups. We employed the Akaike Information Criterion (AIC) to select the optimum number of brain states across the whole movie, defined as the point at which adding more states into the model no longer yielded a better explanation of the variance, as estimated by the free energy. A secondary analysis assessing brain state dynamics during positive scenes was also performed using timeseries extracted from 14 canonical whole-brain networks during the whole movie. This analysis aimed to assess the existence of broader changes in brain state dynamics [16, 44].

To identify differences in the synchronous activity of brain states between melancholic and non-melancholic participants, we counted how many participants visited a particular brain state in sliding time windows around each whole-brain volume (−3 to +3 TR intervals for a total window length of 7 TRs). In line with the above, we only considered time points that aligned with the 22 positive scenes of the movie (Supplementary Table 1). Next, we calculated consistency scores from 0% (no overlap) to 100% (a brain state is present in all participants). A first null model was used to assess whether there was a difference in *between-group* brain state synchronisation. By randomly sampling participants from the whole cohort, we formed 1000 new groups of the same size as the original melancholic and non-melancholic groups. This resulted in 2 × 1000 consistency estimates derived from mixing melancholic and non-melancholic participants. The resulting null distributions were contrasted to the observed brain state consistencies, with the significance threshold set above the 95th percentile. A second null model was also calculated to assess *within-group* brain state synchronisation non-attributable to random fluctuations. ‘Null’ Viterbi paths were generated based solely upon a random initial state and probabilities encoded in the HMM transition matrix belonging to that group. An average consistency between participants in each group were then calculated. This operation was repeated 1000 times to obtain a group-specific null distribution. The 95th percentile determined the threshold above which *within-group* brain state synchronisation was considered significant. Accordingly, up arrows in the relevant figures (Fig. 4; Supplementary Fig. 6) indicate periods where the observed synchronisation was higher than both the between- and the within-group null thresholds.

The analysis of brain state across the positively valenced scenes within the movie also included assessments of (i) state-specific fractional occupancy and dwell time and (ii) individual state transition matrices, encoding for the probability of switching between brain states [16]. We used univariate statistics (two-tailed unpaired *t*-tests, *p*_FWE_ < 0.05) to assess putative between-group differences in fractional occupancy and dwell time. The Network-Based Statistic (NBS) was used to test for group differences in brain state transition probabilities (network-level *p*_FWE_ < 0.05) [45].

### Linking facial movement to brain activity in the positive valence map

We were interested in correlating brain activity recorded (inside the scanner) during positive scenes in the movie with the intensity of facial muscle activity supporting the display of positive affect while viewing a stand-up comedian (outside the scanner). We used SPM12 to model individual fMRI signal in epochs of positive valence during the movie. Specifically, we used a boxcar function with onsets and durations determined by Supplementary Table 1. No convolution with an impulse response model was applied. The resulting single-subject images were used as input into a group-level regression analysis (high threshold of *p* < 0.001, with a following cluster-level correction of *p*_FWE_ < 0.05). For clusters surviving this threshold, the average fMRI contrast values were extracted for each participant. The correlation between fMRI signal and mean facial muscle intensity was investigated using an interaction analysis with ‘*aoctool*’ in Matlab to identify putative differences in this relationship between melancholic and non-melancholic depression.

## Results

### Participants

Seventy participants were recruited and assessed. Forty were classified with non-melancholic and 30 with melancholic depression. The characteristics of the two groups were largely similar (Table 1). Both groups had a relatively high exposure to developmental trauma. As expected, participants with melancholic depression had a higher total MADRS score conferred by neurovegetative and anhedonic symptoms (meaning higher depressive symptoms, mean total MADRS score melancholic = 29.6, mean non-melancholic = 22.0, *t* = −3.3, *p*_FDR_ = 0.01) and a higher total SHAPS score (meaning more anhedonia, mean melancholic = 7.4, mean non-melancholic = 4.2, *t* = −3.4, *p*_FDR_ = 0.01). The significantly greater neurovegetative and anhedonic symptoms in participants with melancholia, as assessed with the MADRS, included reduced sleep, reduced appetite and inability to feel (Table 1). One participant with non-melancholic depression was not able to complete the fMRI component of the study due to claustrophobia.

**Table 1:** Characteristics of the study cohort.

Characteristics	Total (*n* = 70)	Melancholic (*n* = 30)	Non-melancholic (*n* = 40)
Sex (percent)			
Male	28 (40.0)	14 (46.7)	14 (35.0)
Female	42 (60.0)	16 (53.3)	26 (65.0)
Age in years (mean (SD), median (range))	44.0 (±12.8), 46 (24–65)	44.7 (±13.3), 46 (24–65)	43.4 (±12.5), 45 (24–65)
Body mass index (mean (SD), median (range))	28.5 (±6.2), 28 (18–46)	29.0 (±6.5), 28 (18–44)	28.1 (±6.0), 28 (19–46)
Age depression onset (mean (SD), median (range))	18.6 (±9.9), 16 (5–48)	19.9 (±10.3), 17 (5–46)	17.5 (±9.7), 16 (5–48)
Antidepressant trials (mean (SD), median (range))	4.8 (±3.2), 4 (0–13)	5.6 (±3.3), 6 (0–13)	4.2 (±2.9), 4 (1–12)
MADRS (mean (SD), median (range))	25.3 (±10.1), 25 (6–44)	29.6 (±10.1), 34 (8–44)	22.0 (±8.9), 23 (6–42)*
MADRS sleep (mean (SD), median (range))	2.7 (±1.7), 3 (0–6)	3.3 (±1.6), 4 (0–6)	2.2 (±1.7), 2 (0–5)*
MADRS appetite (mean (SD), median (range))	1.1 (±1.6), 0 (0–5)	1.7 (±1.7), 0 (0–5)	0.7 (±1.3), 0 (0–5)*
MADRS feeling (mean (SD), median (range))	2.8 (±1.6), 3 (0–5)	2.8 (±1.5), 3 (0–5)	1.7 (±1.4), 2 (0–5)*
HAM-A (mean (SD), median (range))	20.1 (±8.6), 20 (4–45)	22.2 (±7.6), 23 (4–39)	18.5 (±8.9), 19 (5–45)
SHAPS (mean (SD), median (range))	5.6 (±4.2), 6 (0–14)	7.4 (±4.0), 8 (0–14)	4.2 (±3.8), 3 (0–13)*
Antidepressant use	55	26	29
Fluoxetine equivalent dose (mg) (mean (SD), median (range))	36.1 (±30.6), 40 (0–118)	41.4 (±31.7), 40 (0–118)	31.9 (±29.4), 22.2(0–95.7)
Antipsychotic use	11	7	4
Risperidone equivalent dose (mg) (mean (SD), median (range))	0.2 (±0.7), 0 (0–5)	0.4 (±1.0), 0 (0–5)	0.1 (±0.4), 0 (0–2)
Mood stabiliser use	3	1	2
Benzodiazepine use	4	2	2
Stimulant use	8	4	4
Education level (percent)			
Primary school	1 (1.4)	1 (3.3)	0 (0)
Junior high school	7 (10.0)	3 (10.0)	4 (10.0)
High school	6 (8.6)	4 (13.3)	2 (5.0)
Diploma	11 (15.7)	8 (26.7)	3 (7.5)
University degree	28 (40.0)	8 (26.7)	20 (50.0)
Postgraduate degree	17 (24.3)	6 (20.0)	11 (27.5)
Alcohol use (last 3 months) (percent)			
Never	23 (32.9)	11 (36.6)	12 (30.0)
Once or twice	11 (15.7)	6 (20.0)	5 (12.5)
Monthly	7 (10.0)	0 (0)	7 (17.5)
Weekly	14 (20.0)	7 (23.3)	7 (17.5)
Daily	15 (21.4)	6 (20.0)	9 (22.5)
Tobacco use (last 3 months) (percent)			
Never	61 (87.1)	28 (93.3)	33 (82.5)
Once or twice	2 (2.9)	0 (0)	2 (5.0)
Monthly	0 (0)	0 (0)	0 (0)
Weekly	0 (0)	0 (0)	0 (0)
Daily	7 (10.0)	2 (6.6)	5 (12.5)
Employment status (percent)			
Full time	37 (52.9)	15 (50.0)	22 (55.0)
Part time	9 (12.9)	2 (6.7)	7 (17.5)
Unemployed	22 (31.4)	12 (40.0)	10 (25.0)
Retired	2 (2.9)	1 (3.3)	1 (2.5)
Developmental vulnerabilities (percent)			
Attachment disorder	31 (44.3)	11 (36.7)	20 (50.0)
Physical abuse	14 (20.0)	2 (6.7)	12 (30.0)
Sexual abuse	16 (22.9)	8 (26.7)	8 (20.0)
Bullying	25 (35.7)	12 (40.0)	13 (32.5)
Domestic violence	15 (21.4)	6 (20.0)	9 (22.5)

For all scales, higher scores indicate greater severity of symptoms.

*HAM-A* Hamilton Anxiety Rating Scale (range 0–56), *MADRS* Montgomery-Åsberg Depression Rating Scale (range 0–60), *SHAPS* Snaith-Hamilton Pleasure Scale (range 0–14).

*p_FDR_  <  0.05.

Medication profiles between non-melancholic and melancholic participants were similar (Table 1). Most participants (*n* = 55) were taking antidepressant medication. There was no statistically significant difference in antidepressant dose between groups (mean fluoxetine dose in melancholic participants 41.4 mg (±31.7), mean dose in non-melancholic participants 31.9 mg (±29.4); *t* = 1.50, *p* = 0.20). Only a small number of participants were taking medication from other classes. The small proportion of participants using antipsychotic medication employed atypical antipsychotics at low dose (the mean daily dose of risperidone in both groups was <0.5 mg) and there were no differences between groups (mean risperidone dose in melancholic participants 0.4 mg (±1.0), mean dose in non-melancholic participants 0.1 mg (±0.4); *t* = 1.74, *p* = 0.20). Other forms of psychotropic medication were sparsely employed amongst the cohort and equally distributed between melancholic and non-melancholic groups.

### Facial movement analysis

**Fig. 2:**
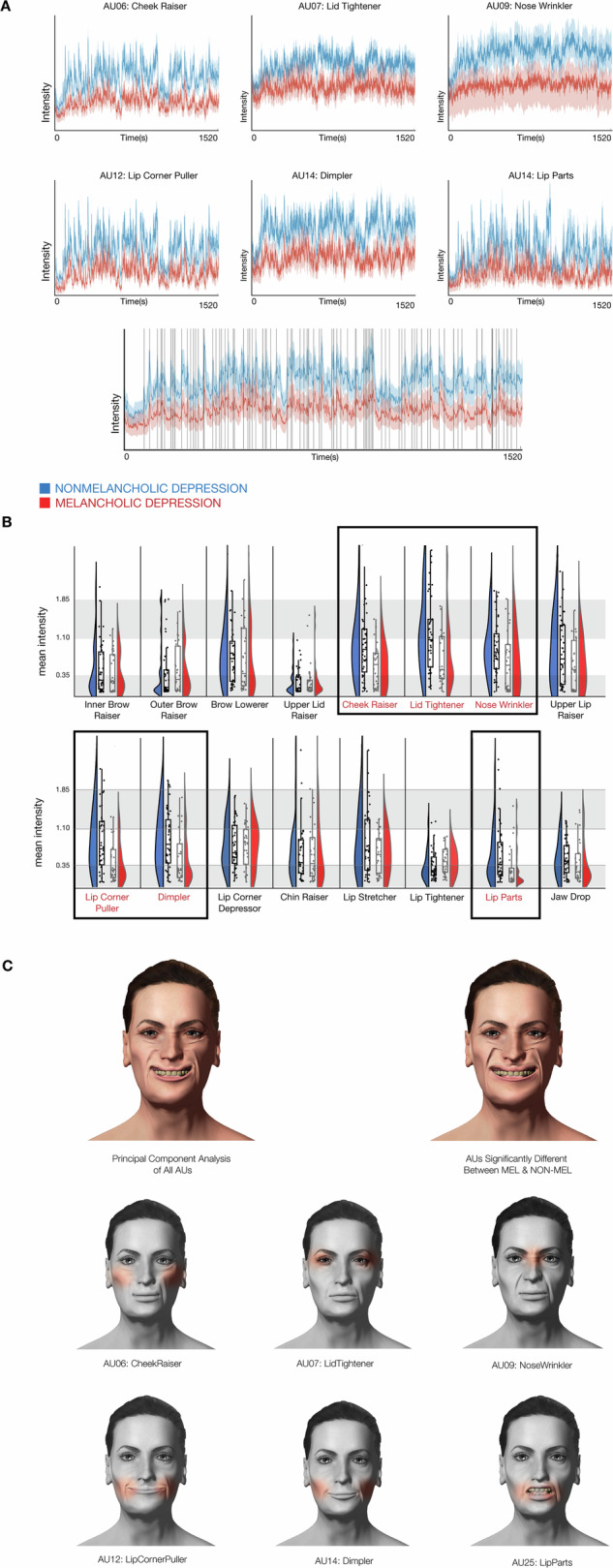
Facial analysis. **A** Timeseries of six facial action units, associated with positive affect, that differ significantly between participants with melancholic (red) and non-melancholic (blue) depression whilst watching a movie clip of a stand-up comedian. Lightly shaded regions indicate the 5th and 95th confidence intervals of the mean. The bottom time series is the first principal component of all 16 facial action units, with vertical lines denoting audience laughter. **B** The violin and box plots depict the median activity of each facial action unit across the whole movie, comparing melancholic (red) and non-melancholic (blue) participants. Action units in red font denote those units with a statistically significant difference in mean activity between melancholic and non-melancholic participants. These action units are also framed for enhanced identification. **C** Representation of facial unit activity on an avatar created using FACSvatar and rendered using FACSHuman (please see main text for citations). In colour, the first principal component of all 16 facial action units (left avatar) and the combined activity of the 6 units that showed a statistically significant difference between melancholic and non-melancholic groups during the comedy viewing (right avatar). This discriminates typical melancholic (inactive) versus non-melancholic (active) responses to stimuli of positive valence. The similarity between these two avatars highlights that the bulk of the variance between melancholic and non-melancholic participants is conferred by the six units contributing to the display of positive affect. Each of the six facial action units differently engaged between melancholic and non-melancholic participants is displayed in greyscale.

There was no significant difference between melancholic and non-melancholic participants in AU 45 (Blink) (*t* = 0.2, *p*_FDR_ = 0.4), and this action unit was not further analysed. Participants with melancholic depression had significantly reduced mean activity in six facial action units (AUs) involved in smiling (Fig. 2A, B, Supplementary Fig. 2, Supplementary Table 3). These were AU06 (Cheek Raiser) *p*_FDR_ = 0.029; AU07 (LidTightener) *p*_FDR_ = 0.030; AU09 (NoseWrinkler) *p*_FDR_ = 0.040; AU12 (LipCornerPuller) *p*_FDR_ = 0.026, AU14 (Dimpler) *p*_FDR_ = 0.029 and AU25 (LipParts) *p*_FDR_ = 0.044.

Using the FACSHuman avatar to precisely represent facial dynamics driven by the activity of each AU, the first principal component of the variance amongst all 16 AUs (Fig. 2C, top left avatar) was compared to that evoked by the combined activity of the 6 facial AU showing a between-group difference (Fig. 2C, top right avatar). The similarity between the facial effects displayed by these two avatars indicates that participants with melancholia demonstrated reduced activity in facial AUs supporting the display of positive affect, with the greatest degree of variance in facial movement conferred by these six AUs. Using an alternative male avatar, the mean activity of facial AUs was compared between melancholic and non-melancholic participants, clearly demonstrating a difference in facial affect between the two groups (Supplementary Fig. 3).

### Changes in brain state synchrony

**Fig. 3:**
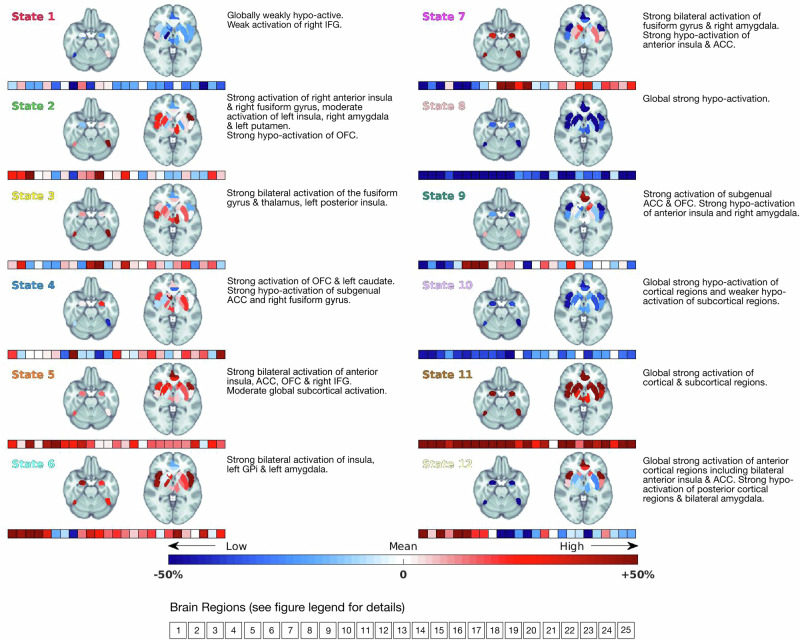
Brain states. A mask comprising brain regions involved in the evaluation of and response to positively weighted stimuli was derived via the neuroimaging meta-analysis tool Neuroquery.org (see section ‘Materials and Methods’). The blue–red colour bar indicates the relative activity loading compared to the average fMRI signal across the whole sample (non-melancholia and melancholia groups). The signal loading across brain regions is also represented in the squares below each brain, from left to right: 1 = left anterior insula, 2 = left posterior insula, 3 = right anterior insula, 4 = right posterior insula, 5 = bilateral superior anterior cingulate cortex, 6 = bilateral pregenual anterior cingulate cortex, 7 = bilateral subgenual anterior cingulate cortex, 8 = bilateral orbitofrontal cortex, 9 = right inferior frontal gyrus, 10 = left fusiform gyrus, 11 = right fusiform gyrus, 12 = right hippocampus, 13 = right amygdala, 14 = right thalamus, 15 = right nucleus accumbens, 16 = right globus pallidus, 17 = right putamen, 18 = right caudate, 19 = left hippocampus, 20 = left amygdala, 21 = left thalamus, 22 = left nucleus accumbens, 23 = left globus pallidus, 24 = left putamen, 25 = left caudate.

Inversion of the HMM using data from 69 participants yielded 12 distinct brain states (Fig. 3, Supplementary Fig. 4). As described above, the loading of brain states onto 14 canonical networks was also calculated (Supplementary Fig. 5) [44].

**Fig. 4:**
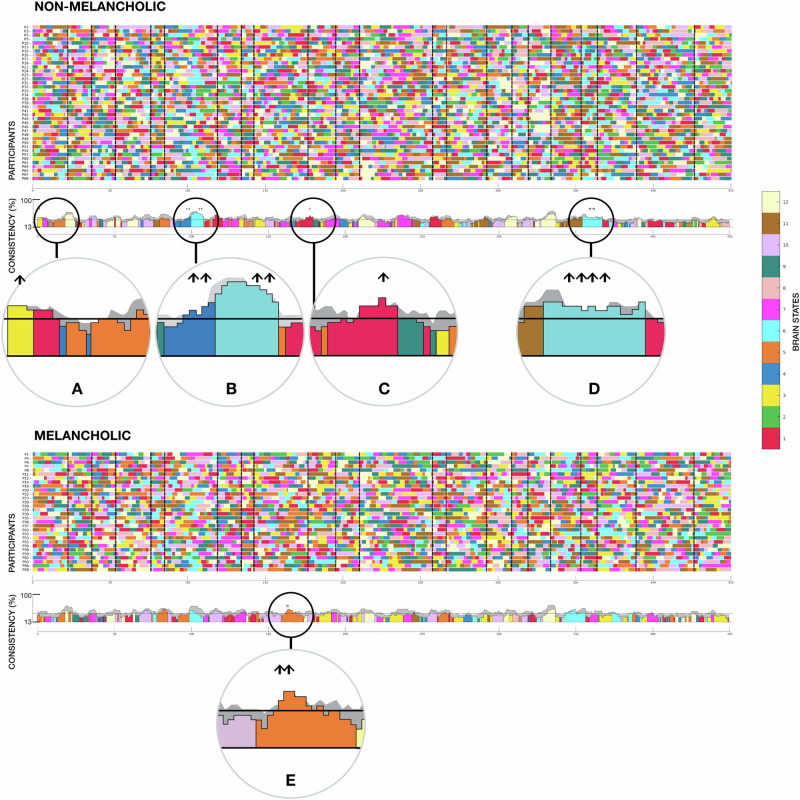
Brain state dynamics during movie viewing. Brain state dynamics during viewing of an emotionally evocative movie for non-melancholic participants (upper panel) and melancholic participants (lower panel). Brain states are colour-coded according to the legend on the far right (mirroring Fig. 3, showcasing brain state topology). The temporal consistency across participants is presented as a timeline in the lower aspect of each panel. The black horizontal line represents the within-group state synchronisation based on the null model. The grey-shaded region represents the between-group null distribution (section ‘Materials and Methods’). Periods during which group synchrony is greater than the top 5% of these two null distributions are represented by an upwards-facing arrow. Periods of exended group synchrony for the non-melancholic group are **A** scene 1: state 3, **B** scene 6: states 4 and 6, **C** scene 10: state 1 and **D** scene 19: state 6. Periods of protracted group synchrony for the melancholic group are **E** scene 9: state 5. Further details of these scenes and group differences can be found in Supplementary Table 1.

We then assessed the degree of inter-subject consistency in the expression of the 12 brain states whilst participants watched positively valenced scenes. To accomplish this, we specified pre-defined regions of interest in a ‘*positive valence map*’ via a meta-analysis of existing neuroimaging data. We identified synchronous brain states amongst participants and calculated the proportion of participants sharing a brain state at a given epoch with the movie, yielding a consistency metric. Compared to prior work investigating whole-brain dynamics [16] with high weightings in visual, auditory and language networks, participants’ expression of states within our positive valence brain map was more heterogeneous. Nevertheless, visualisation of the temporal consistency across all participants identified discrete periods of greater above-null synchronous activity within states 1 (red), 3 (yellow), 4 (dark blue) and 6 (light blue) amongst non-melancholic participants (Fig. 4, upper panel, Supplementary Table 1) as compared to melancholic participants (Fig. 4, lower panel, Supplementary Table 1). Conversely, melancholic participants displayed greater above-null synchrony of state 5 (orange) than non-melancholic participants. These results highlight higher synchronous activity in the left amygdala and reduced activity in the subgenual anterior cingulate in non-melancholic participants during the appraisal of affective stimuli. At the same time, melancholia mapped onto higher synchronous activity of core salience regions (anterior insula and anterior cingulate cortex). These changes in between-group state synchrony arose in discrete periods within the narrative architecture of the movie that were unified by uplifting and pleasantly surprising plot evolutions (described in Fig. 4 legend and Supplementary Table 1).

Synchrony across the whole cohort was greater when employing masks of whole-brain canonical networks (Supplementary Figs. 5 & 6). While these broader masks revealed fewer differences between melancholic and non-melancholic participants (Supplementary Fig. 6), they support changes in the engagement of the salience, executive and default mode networks between the two groups. These results are in line with the findings derived from our more spatially restricted, predominantly frontal-subcortical ‘*positive valence map*’.

No between-group changes were detected in fractional occupancy, dwell time and brain state transitions.

### Covariation of facial movement and brain activity

**Fig. 5:**
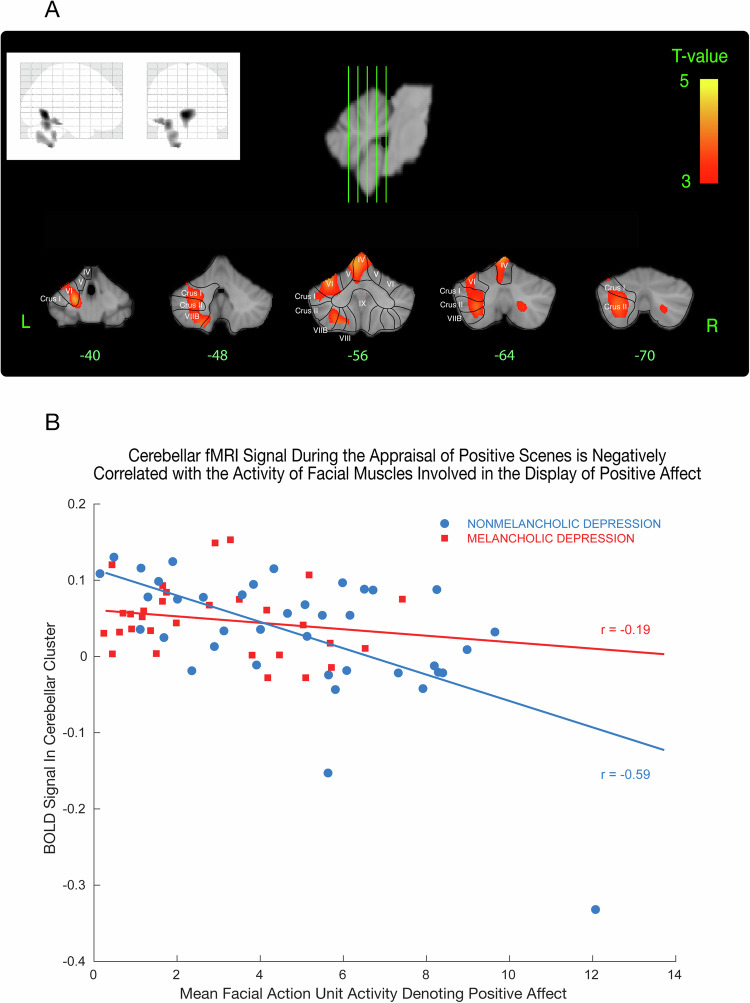
Cerebellar clusters covary negatively with mean positive facial action unit activity. **A** Two clusters in the cerebellum that covaried negatively with mean positive facial action unit activity (i.e., the higher the activity of facial muscles involved in the display of positive affect, the lower the fMRI signal in these clusters). **B** The largest cluster was found in the left posterior cerebellar hemisphere, where we detected a significant difference in this relationship between participants with melancholic and non-melancholic depression (analysis of covariance *F* = 4.11, *p* = 0.047). Clinical characteristics and data quality were reviewed for all participants and there were no discrepancies amongst variables such as clinical status, medication use, head motion, normalisation or whole-brain coverage for any participants. All data points were within 2.5 standard deviations of the group mean for melancholic and non-melancholic participants.

We identified two clusters in the cerebellum (Fig. 5A, Supplementary Table 4) that covaried negatively with mean positive facial action unit activity (i.e., the higher the activity of facial muscles involved in the display of positive affect, the lower the activity in these clusters). The largest cluster comprised lobules VI and VII and a portion of the cerebellar crus in the left cerebellar hemisphere. There was a significant difference in the relationship between participants with melancholic and non-melancholic depression and cerebellar activation in this cluster (*F* = 4.11, *p* = 0.047), with melancholic participants displaying a more blunted relationship between cerebellar activity and facial action unit activity during positive movie scenes (Fig. 5B).

### Other symptom dimensions

To examine the specificity of our findings to depression amongst participants with melancholia, we examined whether facial and brain state dynamics were also influenced by anxiety symptoms. After controlling for multiple comparisons, no statistically significant associations existed between the mean activity of the six facial AUs involved in smiling and the severity of anxiety symptoms as rated with the HAM-A (Supplementary Fig. 7A). There was also no statistically significant relationship between the occupancy of brain state 5, which differentiated melancholic from non-melancholic participants, and anxiety symptoms (Supplementary Fig. 7B).

## Discussion

In participants with depression recruited from a unique melancholia-enriched cohort, we studied facial and brain state dynamics during the processing of positively weighted stimuli. Based on the central role of psychomotor retardation and anhedonia in the melancholic phenotype, we hypothesised that participants with melancholia would display a significant reduction in facial expressivity in response to positive stimuli. We also expected a core difference in brain activity in response to stimuli of positive valence, related to the attenuation of affect in melancholia. Results support both hypotheses and the distinction between melancholic and non-melancholic depression. We observe robust statistical differences between groups in both facial expressions and brain state dynamics during the appraisal of video scenes characterised by positive emotional tone. These findings are important because they suggest that melancholia differentially affects, relative to non-melancholic depression, the neural substrate responsible for processing ecologically valid multisensory stimuli and that this change manifests at the motoric level (i.e., facial muscle activity).

While viewing a comedy clip, participants with melancholia had significantly reduced activity in six facial action units underpinning the display of positive affect, which was detected and tracked with a convolutional neural network. These findings extend previous work examining facial dynamics in depression that has compared participants with melancholia with non-depressed controls [18] as well as the automatic detection of depression from facial expressions, eye contact, and head movements [46–48]. Whereas previous work has been based upon an agnostic, classifier-based distinction, our findings offer more insight into the underlying nature of this difference. They suggest not only that melancholic and non-melancholic depression can be effectively subtyped but also that impaired affective reactivity to scenes of positive valence is a pronounced feature of melancholic as compared to non-melancholic depression.

Whilst a direct comparison between non-depressed healthy controls and participants with both melancholic and non-melancholic depression is absent here, our findings align with previous research that demonstrates that melancholia is characterised by a generalised blunting of affective reactivity [18] and that participants with non-melancholic depression are more like controls in this domain. Future work may include participants with other affective disorders, such as bipolar depression or schizoaffective disorder, to determine the specificity of our findings and whether there are brain and behavioural signatures that differentiate these depressive subtypes.

We observe robust statistical differences between groups in both facial expressions and brain state dynamics during the appraisal of video scenes characterised by positive emotional tone. These findings are important because they suggest that melancholia differentially affects, relative to non-melancholic depression, the neural substrate responsible for processing ecologically valid multisensory stimuli and that this change manifests at the motoric level (i.e., facial muscle activity).

Brain states occurring during movie viewing were estimated using the HMM, under the model that the observed data arise from a smaller number of hidden states and their transitions. The expression of brain states implicated in detecting and processing positive stimuli differed between melancholic and non-melancholic participants. Finally, in converging evidence of altered processing of positive stimuli in melancholia, only in non-melancholic depression could the display of positive affect in the facial landmark-tracking paradigm be strongly associated with the activity of two clusters within the cerebellum, measured during positively weighted excerpts of the movie shown within the scanner.

Overall, we find multimodal evidence of altered processing of positive stimuli in melancholic as compared to non-melancholic depression. These results further contribute to quantitative psychophysical and neural evidence in support of the clinical perspective that persons with melancholia display a characteristic phenomenology marked by pronounced psychomotor retardation (reduced facial muscle activity underpinning a reduction in positive affect) and profound anhedonia (differences in expressed affect are seen in response to a comedy clip whilst differences in brain state activation emerge during positively weighted movie scenes). We sought to connect brain state transitions with dynamic markers of facial affect, linking these motoric and central evaluative domains in melancholia. In the cerebellum, we find a neural substrate through which differences in facial dynamics may be expressed.

The cerebellum’s contribution to ‘*higher order*’ neuropsychological constructs such as executive functioning and affect regulation has long been recognised [49]. Whilst the anterior lobe of the cerebellum is principally engaged in motor control, the cerebellar vermis and posterior lobe contribute to affective processing and complex cognitive operations [50]. The role of the posterior lobe, in this context, may be to ‘*fine-tune*’ emotional tone [51]. The cerebellum is bidirectionally connected to other brain regions implicated in processing socially and emotionally salient stimuli, such as the prefrontal cortex, the amygdala and the septal nuclei, providing it with the computational tools to fulfil this role. Lesions of the posterior lobe and vermis of the cerebellum have previously been associated with an impairment in the subjective experience of pleasant feelings in response to happiness-evoking stimuli [52], whilst a variety of different human emotions evoke spatially distinct patterns of activity in the posterior lobe [53]. In a meta-analysis of neuroimaging studies examining the topographic arrangement of affective processing in the cerebellum, lobules VI, VII and Crus I were encompassed by activation likelihood estimate clusters similar to our findings, with the largest clusters in the left cerebellar hemisphere [54]. Consistent with the crossed cerebro-cerebellar fibre pathways, it is of interest that we also find our largest cluster in the left posterior cerebellar lobe, given the established right-hemispheric predominance of ‘*cognitive-control*’ operations such as inhibition in regions including the subthalamic nucleus, inferior frontal gyrus and supplementary motor area [55–58]. Recent work has identified a disynaptic anatomical pathway between the posterior cerebellum and the forebrain mediating these ‘*non-motor*’ functions [59] and the same movie employed in our investigation has previously been shown to engage the posterior cerebellum during key narrative shifts [60]. An outstanding question is why we observe a negative correlation between cerebellar activity and affective facial dynamics in non-melancholic depression but not in people with melancholia. This could be related to the emotional ‘*blunting*’ often observed in melancholia, potentially representing inefficient emotional processing in this group.

We modelled the expression of dynamic configurations of brain states and found that, amongst participants with non-melancholic depression, state 6 emerges above the null distributions at key narrative junctures within the movie marked by positive surprise (e.g., unexpected kindness towards the protagonist and the protagonist’s survival after a near-drowning). State 6 is characterised by strong activation of the amygdala and insula, regions supporting the affective processing of stimuli. Amongst melancholic participants, only state 5 supersedes the nulls at one point in the movie, again a moment of surprise (the protagonist is found stowed away by the circus). State 5 comprises cortical nodes of the salience network, centred upon the anterior insula and the dorsal anterior cingulate cortex [61]. This network supports dynamic shifts in attention from an interoceptive to an externally oriented focus [62]. Previous work has demonstrated disrupted effective connectivity amongst nodes in this network in people with melancholia, mediating the internally dysphoric, ruminative thinking style characteristic of this subtype of depression [10]. The consistent engagement of this network at this point in the narrative architecture of the movie amongst melancholic participants may represent the inefficient ability to assign salience and effectively shift attention to surprising plot shifts.

One notable finding from our investigation is that the dynamics of facial affect appear more sensitive in differentiating melancholic from non-melancholic depression than the dynamic expression of functional brain states. For two reasons this is foreseeable. First, both cohorts are unified by their core diagnosis of depression, which regardless of the subtype, will influence the emotional tone applied to the perception of a complex stimulus such as a movie. Second, facial expressivity is the manifestation of a downstream effector reflecting not just brain state in our focused regions of interest, but also the interaction and integration of this ‘*positive valence map*’ with sensorimotor and associated supplementary brain regions. Indeed, we also observed group differences in the expression of brain states defined by large-scale brain networks during the appraisal of positive movie scenes. Therefore, it may only be at this ‘*end-organ’* stage that manifest differences between the two phenotypes of depression clearly arise. Finally, fMRI has an poorer time resolution compared to video recording, which may render it less sensitive to detecting between-group fluctuations at high temporal frequencies. Future work could employ intracranial electrophysiological recordings, such as, for example, during the implantation of permanent deep brain stimulation electrodes, to better study the relationship between brain activity and facial expression. Future work may also leverage cross-sectional ratings of the momentary feeling state of participants, in order to identify correlations with subjective emotions, brain activity and facial expressivity.

One limitation of the study is that melancholic participants did have a (small but statistically significant) higher depressive (MADRS) rating scale score than non-melancholic participants. However, this difference was driven by neurovegetative symptoms (anorexia, insomnia) and anhedonia, consistent with the core phenotype of melancholia. It is also important to note that our study is not powered to solve the question of whether melancholia should be viewed dimensionally (a more severe expression of depression) or categorically (a distinct subtype). Others have argued for the latter based on the occurrence of specific symptoms and signs, a greater relevance of biological rather than social factors and a selective response to physical treatments [3, 7, 9, 27, 28, 63–66].

A further potential limitation is that dynamic changes in facial affect and brain state transitions were examined across two different stimuli (a clip of a stand-up comedian and an emotionally evocative movie). Ideally, facial movement analysis and functional MRI acquisition should occur simultaneously. However, in our pilot testing, we found that facial landmark detection was obstructed in the cramped confines of the scanner. The finding that we were nonetheless able to link brain dynamics and facial action unit activity through the cerebellum is a relative strength, given that these two streams of data arose from different stimuli, arguably adding to the reliability of our conclusions. Future work may investigate differential patterns of structural cerebellar-cortical connectivity between melancholic and non-melancholic depression, which may be associated with these functional changes.

A final limitation relates to the feasibility of applying these methods to broader clinical populations. Participants recruited for this investigation had already enroled in an existing genetic study and may, therefore, have been a more motivated cohort, especially as the study procedures in this investigation were relatively lengthy (taking a half day). Further work in other populations, such as hospital inpatients, may strengthen the reliability of these findings. Naturalistic stimuli, particularly the comedy clip we employed, are culturally embedded and hence sensitive to cultural and demographic norms. Thus, our clips may not have been appropriate for a very elderly or non-Western ethnic group. However, appropriate replacement film clips for alternative demographic groups could be selected to allow a deeper understanding of the observed effects. Finally, methodological advances are required to translate the adopted approaches from the research to the clinical setting. The complex behavioural and neuroimaging analyses employed here could be simplified for deployment in the hospital or outpatient clinic. A feasible first step would be to focus on facial movement analysis, tailored to various demographic and ethnic settings.

Antidepressants, antipsychotics, benzodiazepines and mood stabilising medication may have varied effects upon facial expressivity and brain state dynamics. Whilst in our study there were no significant differences in medication use between groups, this will be an important factor to consider in future work using similar paradigms. Future work may also acquire biological data from the endocrine and immune systems (such as cortisol levels and inflammatory markers) to gain a broader neurobiological perspective on the differences between depression subtypes [67, 68]. Although our sample comprised participants with a diagnosis of unipolar depression, with major psychiatric comorbidities excluded during a clinical assessment, prospective work could include participants with bipolar depression, schizoaffective disorder and other comorbidities including eating disorders and substance use disorders. Comorbidities such as these may give rise to their own unique brain and behavioural signatures that may prove useful for phenotyping and prognostication. Finally, future work should also assay these variables against a baseline of non-depressed controls, in order to obtain a ‘normative’ sample of data.

Finally, our methodology employed a cross-sectional analysis of our participants, potentially missing the dynamic fluctuations in brain state and affect that characterise a typical depressive course. Future longitudinal studies will clarify whether the changes seen in melancholia are transitional or evolve in response to treatment. Our findings motivate future work assessing if the defined facial and neurophysiological metrics represent new, reliable, and specific prognostic markers of treatment response (e.g., to neuromodulation). The non-invasive and portable nature of facial activity analysis support the feasibility of this endeavour.

In sum, participants with a melancholic phenotype of depression displayed quantitative deficits in facial action unit activity underpinning the display of positive affect during exposure to a stand-up comedian, as compared to participants with a non-melancholic phenotype. At a neural level, the synchronous expression of brain states supporting the experience of positive emotions differed amongst participants with melancholia, as compared to those with non-melancholic depression. Activity in the posterior lobe of the cerebellum may connect these findings, being a means through which brain state dynamics influence the external manifestation of positive emotions (laughter, smiling). The primary implication of these findings is their relevance to phenotyping and treatment selection. As interest grows in ‘*precision medicine*’, our results offer a means through which subtypes of the broad depressive construct can be more sensitively and robustly detected. This will have important implications for the personalisation of treatment regimens and, in particular, the stratification of treatment by endophenotype. For example, we have previously hypothesised that one of the factors contributing to the limited success of deep brain stimulation for refractory depression in clinical trials has been a failure to enrich these trials for individuals with melancholia [69]. A more nuanced understanding of the pattern of brain network dysfunction in melancholia may also guide more effective targeting of stimulating electrodes [70], or refinement of the positioning of the stimulating coil in transcranial magnetic stimulation [71]. A pattern of brain network activity or facial muscle activation suggesting a non-melancholic rather than melancholic depressive disorder may also prompt prioritisation of psychotherapeutic and social interventions before biological strategies. Clincal trials of neuromodulation may specifically employ the strategies we detail in this investigation, to enrich experimental cohorts.

## Supplementary information

Supplementary Information

## Data Availability

Deidentified participant data are available from the corresponding author. Movie clips can also be accessed by contacting the corresponding author. The Openface toolbox for Facial Action Unit recognition is available at https://github.com/TadasBaltrusaitis/OpenFace. Scripts to run the HMM analyses are provided at: https://github.com/clinical-brain-networks/HMM-Melancholia.

## Code availability

Scripts to run these analyses are provided at: https://github.com/clinical-brain-networks/HMM-Melancholia. Movie clips can be accessed by contacting the corresponding author.
