# Supplementary material for: Markers of positive affect and brain state synchrony discriminate melancholic from non-melancholic depression using naturalistic stimuli
Source: Mol Psychiatry. 2024 Aug 27;30(3):848–60. doi: 10.1038/s41380-024-02699-y (PMC11835748; doi:10.1038/s41380-024-02699-y)
Supplement: Supplementary file 1 — Supplementary Information [file 41380_2024_2699_MOESM1_ESM.docx]

**SUPPLEMENTARY INFORMATION**

**MARKERS OF POSITIVE AFFECT AND BRAIN STATE SYNCHRONY DISCRIMINATE MELANCHOLIC FROM NON-MELANCHOLIC DEPRESSION USING NATURALISTIC STIMULI**

Philip E. Mosley FRANZCP PhD ^1,2,3,4^, Johan N. van der Meer PhD ^1,5^, Lachlan Hamilton ^1^, Jurgen Fripp PhD ^3^, Stephen Parker FRANZCP PhD ^4,6^, Jayson Jeganathan MBBS ^7,8,9^, Michael Breakspear FRANZCP PhD ^7,8,9^, Richard Parker PhD ^1^, Rebecca Holland B.Des ^1^, Brittany L Mitchell PhD ^1^, Enda Byrne PhD ^10^, Ian B Hickie MD FRANZCP ^11^, Sarah E Medland PhD ^1,12,13^, Nicholas G Martin PhD ^1^, Luca Cocchi PhD ^1,4^

*^1^ QIMR Berghofer Medical Research Institute, Herston, Queensland, Australia*

*^2^ Queensland Brain Institute, University of Queensland, St Lucia, Queensland, Australia*

*^3^ Australian eHealth Research Centre, CSIRO Health and Biosecurity, Herston, Queensland, Australia*

*^4^ Faculty of Medicine, School of Biomedical Sciences, University of Queensland, St Lucia, Queensland, Australia.*

*^5^ School of Information Systems, Queensland Univerisity of Technology, Kelvin Grove, Queensland, Australia*

*^6^ Department of Psychiatry, Royal Brisbane & Women’s Hospital, Herston, Queensland, Australia*

*^7^ School of Psychology, College of Engineering, Science and the Environment, University of Newcastle, Newcastle, New South Wales, Australia*

*^8^ Hunter Medical Research Institute, Newcastle, New South Wales, Australia*

*^9^ School of Medicine and Public Health, College of Medicine, Health and Wellbeing, University of Newcastle, Newcastle, New South Wales, Australia*

*^10^ Child Health Research Centre, University of Queensland, South Brisbane, Queensland, Australia*

*^11^ Brain and Mind Centre, University of Sydney, Camperdown, New South Wales, Australia*

^12^ *School of Psychology, University of Queensland, St Lucia, Queensland, Australia*

*^13^ School of Psychology and Counselling, Queensland Univerisity of Technology, Kelvin Grove, Queensland, Australia*

Correspondence to:

Dr Philip E Mosley, QIMR Berghofer Medical Research Institute, 300 Herston Road, Herston, Queensland, 4006, Australia

Email: philip.mosley@qimrberghofer.edu.au Telephone: +61733620222

Running Title: Facial Movement and Brain State Dynamics in Melancholia

**TABLE OF CONTENTS |**

**FIGURE S1** Page 3

**FIGURE S2** Page 5

**FIGURE S3** Page 6

**FIGURE S4** Page 7

**FIGURE S5** Page 8

**FIGURE S6** Page 9

**TABLE S1** Page 11

**TABLE S2** Page 13

**TABLE S3** Page 14

**TABLE S4** Page 15

**SUPPLEMENTARY FIGURE 1**

The positive valence map identified from the NeuroQuery meta-analysis tool using the terms ‘*feeling, happy, positive, satisfaction, valence, pleasure*’. The whole brain map is depicted in blue. We parcellated this map into 25 subregions using Talairach Daemon (11 cortical regions) and the Melbourne subcortex atlas (14 subcortical regions). Each region overlaps with the NeuroQuery positive valence map and is shown in yellow (four axial slices are shown). See also Supplementary Table 2.


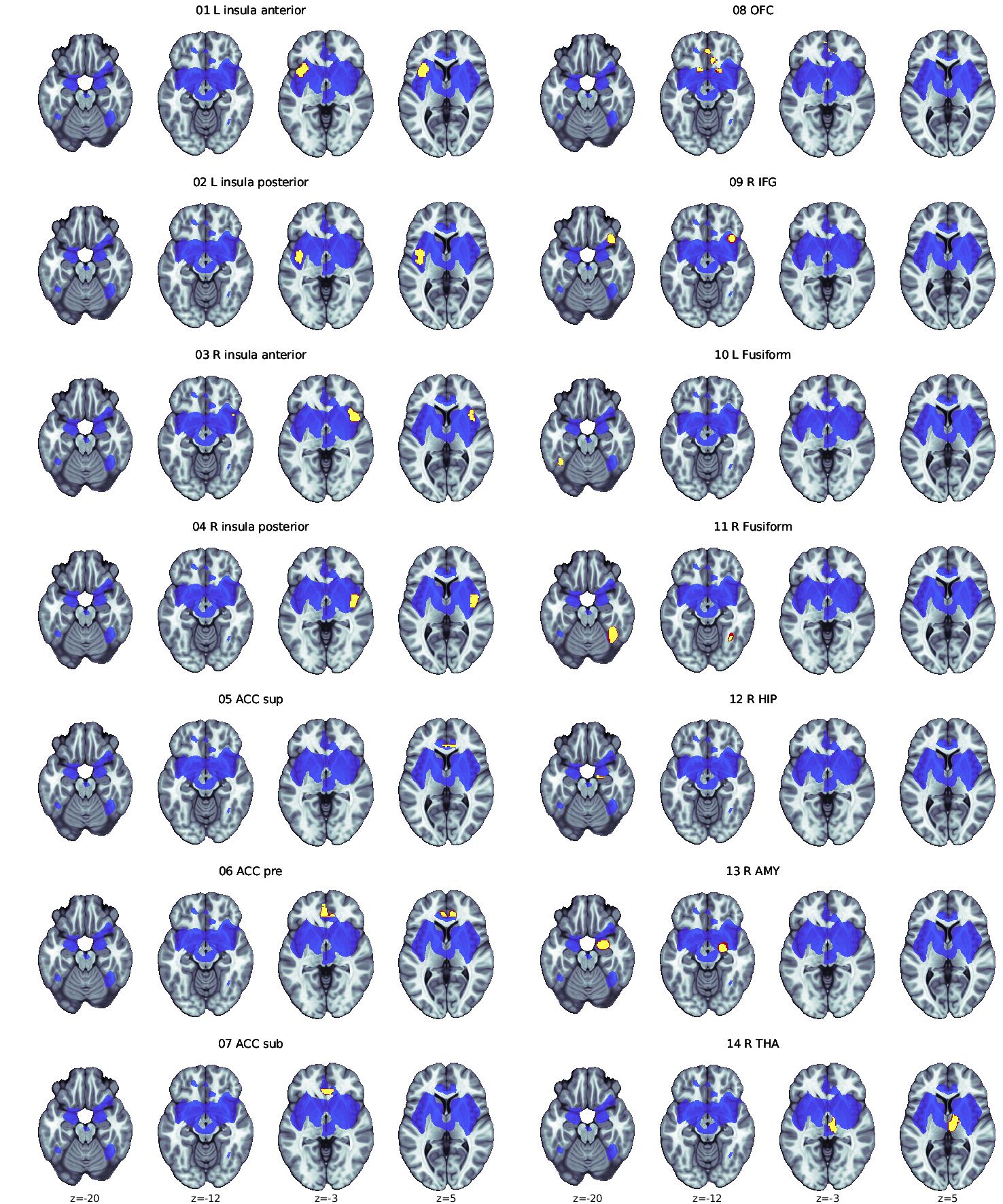


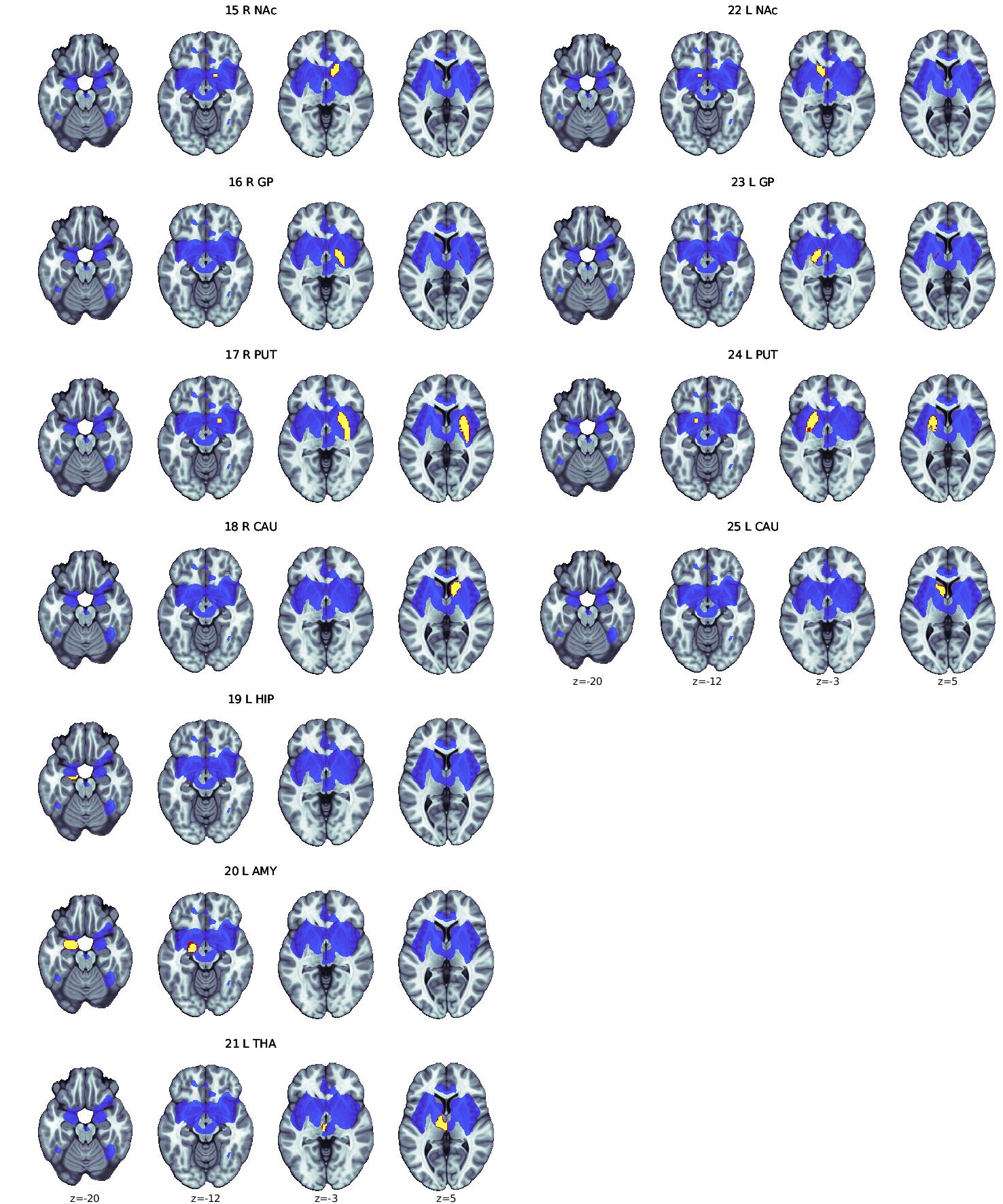


**SUPPLEMENTARY FIGURE 2**

Summed mean activity in facial action units (AUs) during the viewing of a comedy clip, demonstrating a marked reduction in facial expressivity amongst participants with melancholic depression.


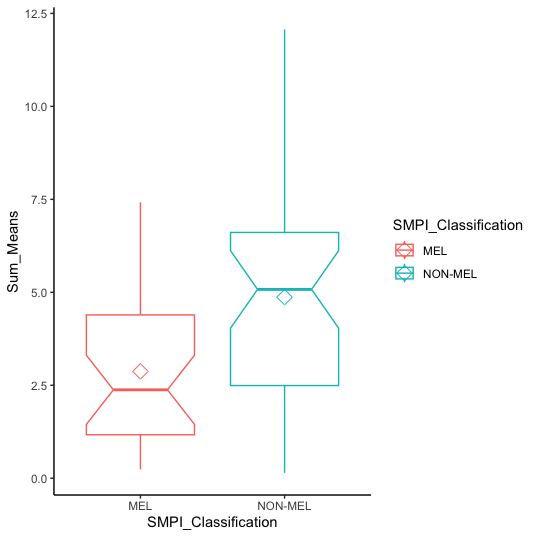


**SUPPLEMENTARY FIGURE 3**

Using a male avatar and the mean activity of facial action units amongst melancholic and non-melancholic participants, the typical facial response of melancholic and non-melanchlic depressed participants to a humorous stimulus is presented, clearly demonstrating a marked reduction in positive affect amongst melancholic partiicpamts.

**
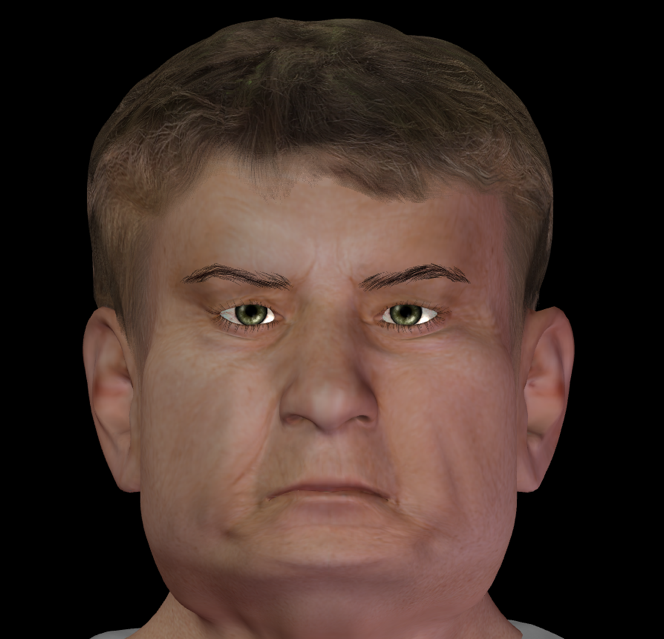
**

**Melancholic**

**
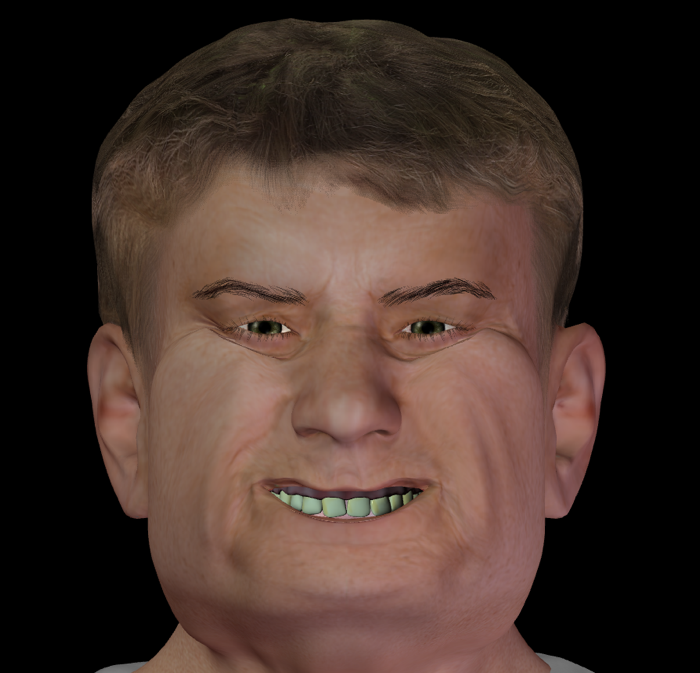
**

**Non-Melancholic**

**SUPPLEMENTARY FIGURE 4**

Evaluating the AIC criterion. With an increasing number of inferred states, the explained free energy of the HMM model decreases. After 12 states (K), increasing the number of states no longer yields a marked decrease in free energy (FE).


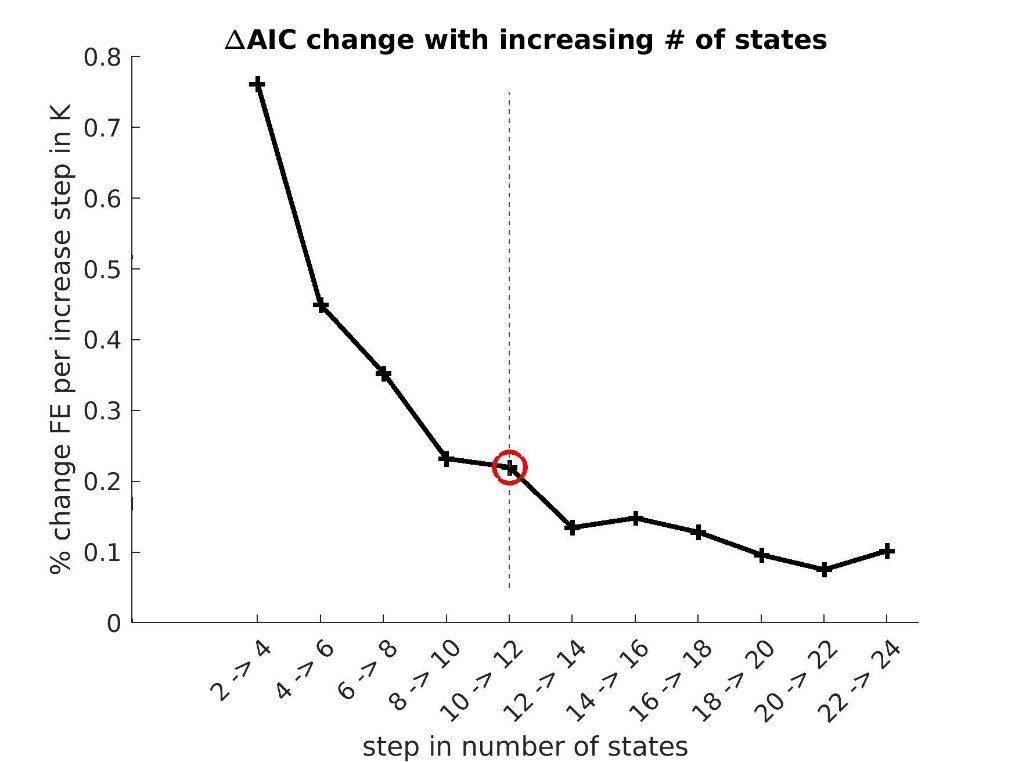


**SUPPLEMENTARY FIGURE 5**

The relative loading of fMRI signal in brain states defined by maps of 14 canonical whole-brain networks: dorsal and ventral Default Mode Networks (dDMN and vDMN), Precuneus (PRE), Anterior Salience Network (ASN), Posterior Salience Network (PSN), Left and Right Executive Control Networks (lECN and rECN), Basal Ganglia Network (BGN), Auditory Network (AUD), Primary Visual Network (pVIS), High Visual Network (hVIS), Sensorimotor Network (SMN), Visuospatial Network (VSN), and Language Network (LAN). These maps can be collapsed into four broader systems: DMN (Default Mode Network), SAL (Salience Network), EXEC (Executive Network), and SENS (Sensory Network). The blue-red colour bar indicates the relative loading compared to the average activity across the cohort used to infer the Hidden Markov Model.


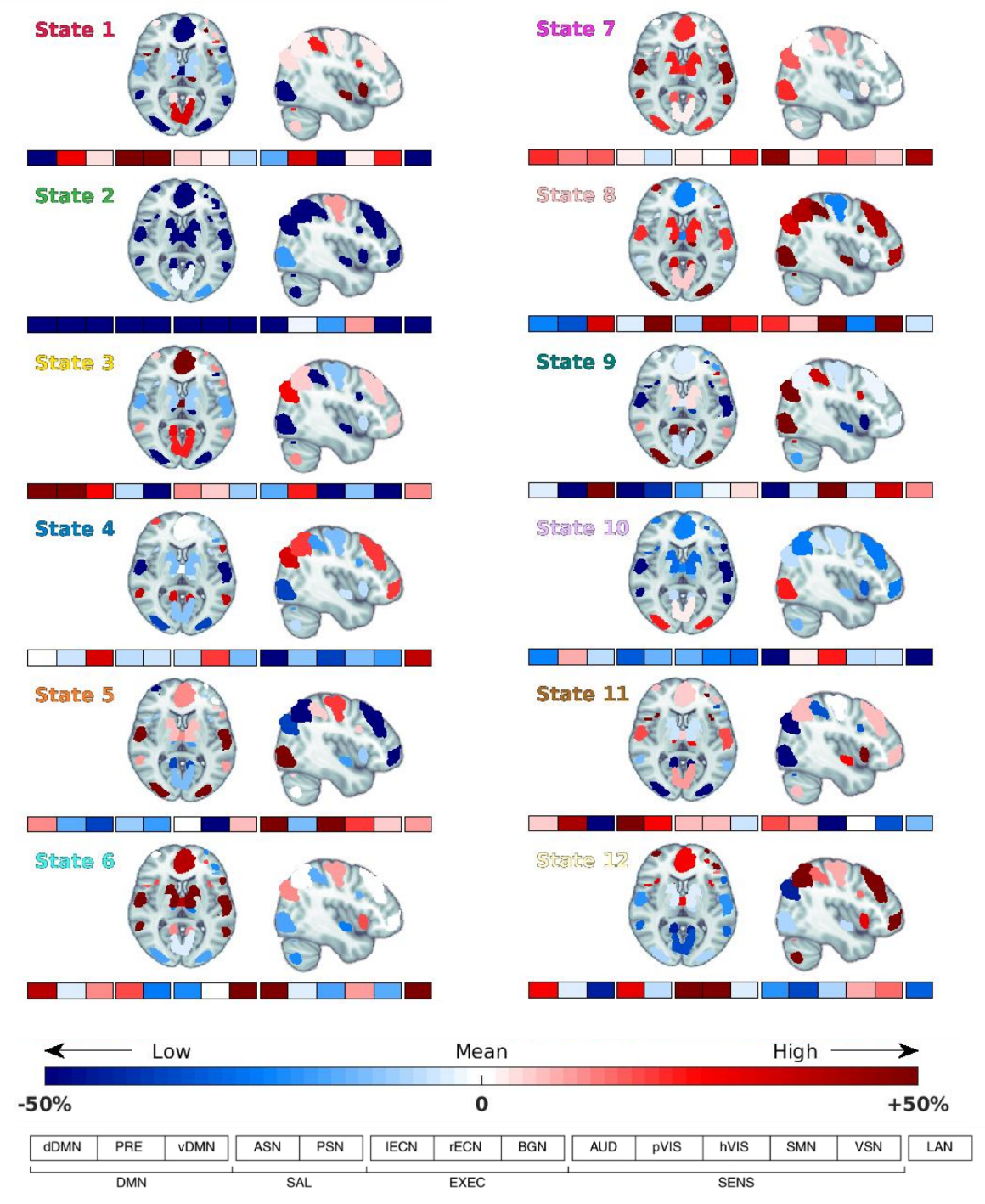


**SUPPLEMENTARY FIGURE 6**

Whole-brain state dynamics while viewing an emotionally evocative movie: non-melancholic participants (upper panel) and melancholic participants (lower panel). Brain states are colour-coded according to the legend on the far right. These brain states are detailed in Supplementary Figure 5 above.


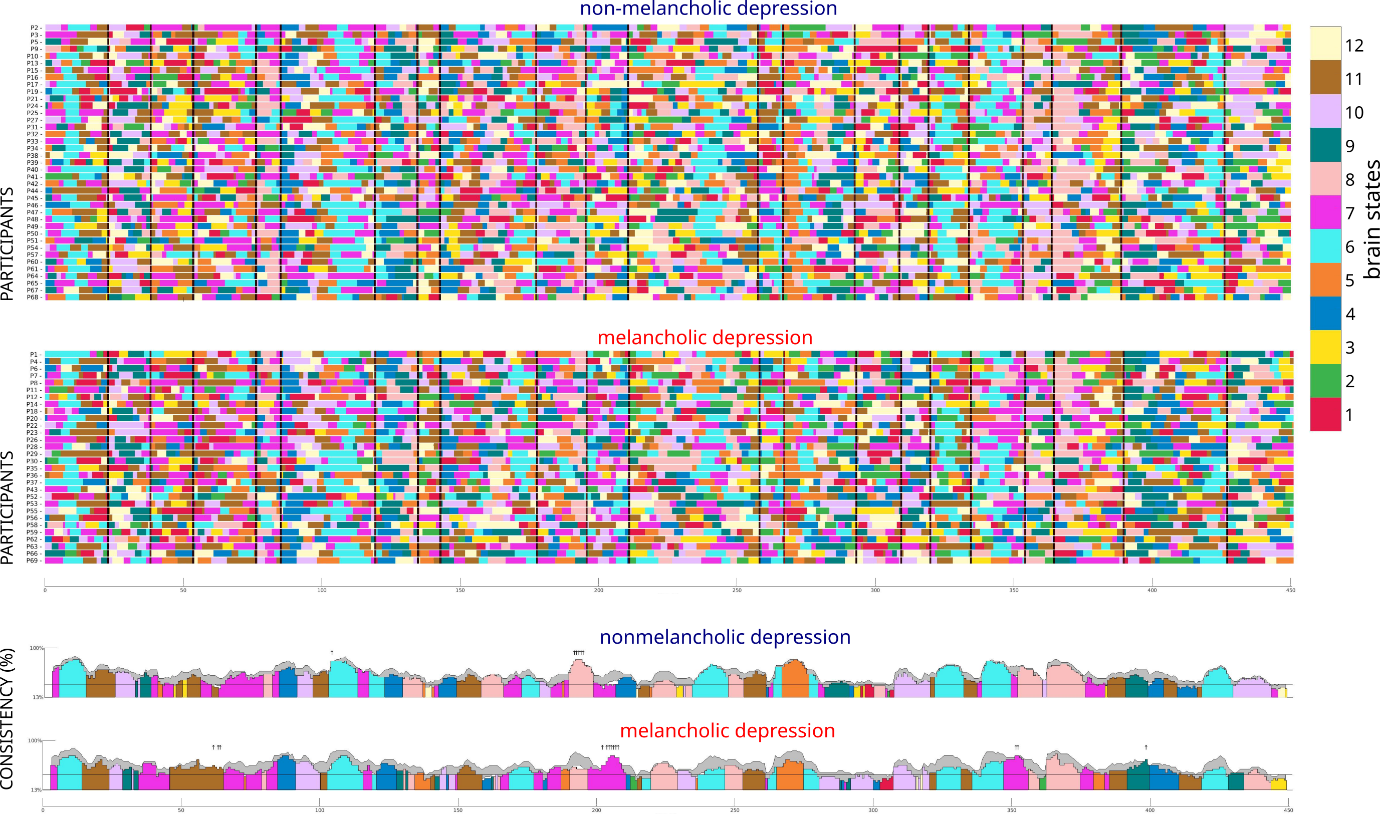


**SUPPLEMENTARY FIGURE 7**

To examine the specificity of our findings to depression, amongst participants with melancholia, we examined whether facial and brain state dynamics were also influenced by anxiety symptoms. Raw values are presented here, but after controlling for multiple comparisons (false discovery rate approach, FDR), there were no statistically significant associations between the mean activity of the six facial AUs involved in smiling and anxiety symptoms as rated with the HAM-A. In the HMM, there was no statistically significant relationship between the above-null synchrony of state 5, observed amongst melancholic participants, and anxiety symptoms.

**
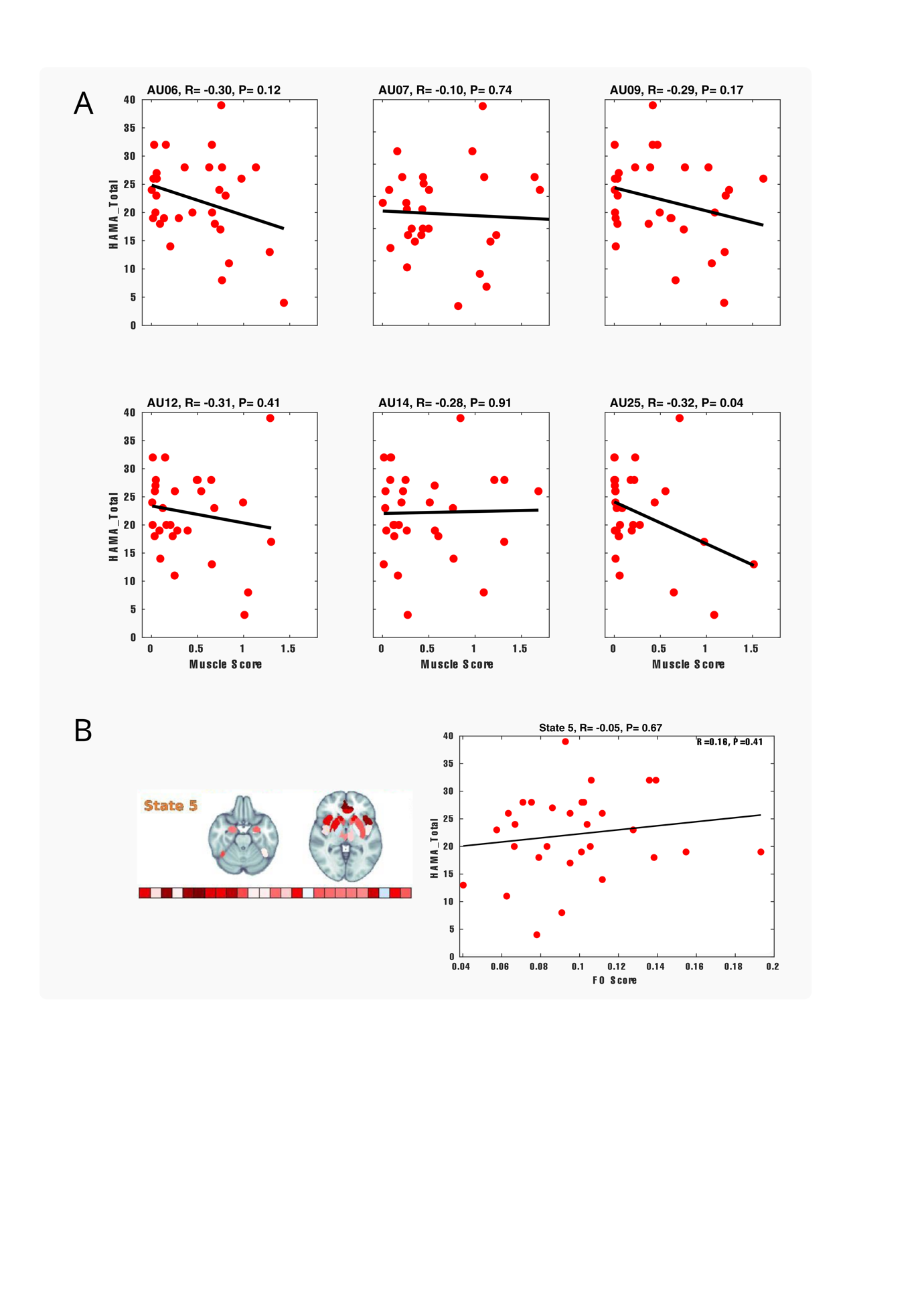
**

**SUPPLEMENTARY TABLE 1**

Positive scenes within the movie (‘The Butterfly Circus’) and brain state synchrony amongst participants with melancholic and non-melancholic depression

| **Period**  **Time (min:sec)** | **Scene Description** | **Enhanced Synchrony Amongst MEL / NON-MEL** |
| --- | --- | --- |
| 1  00:53 – 01:13 | A young boy sees an advertisement for a carnival sideshow. He asks excitedly if he can attend. Mendez (the circus showman) agrees. | NON-MEL increased consistency of state 3. |
| 2  01:20 – 01:33 | The troupe attend the carnival, and everyone is excited. | Nil |
| 3  02:01 – 02:15 | The troupe are encouraged to enter the ‘Freak Show’ | Nil |
| 4  02:52 – 03:14 | The host of the sideshow introduces the exhibits and excitedly prepares the crowd to view the ‘Limbless Man’ | Nil |
| 5  03:45 – 03:53 | Mendez steps in to prevent two boys teasing Will (the Limbless Man) by throwing an apple at him | Nil |
| 6  04:01 – 04:35 | Mendez is kind to Will even though at first Will behaves in a hostile manner | NON-MEL increased consistencies of states 4 and 6. |
| 7  05:36 – 05:49 | Will escapes with the circus and joins the troupe, fooling the host of the sideshow | Nil |
| 8  06:19 – 06:25 | The troupe relax & party together | Nil |
| 9  06:39 – 07:12 | The whole troupe find Will stowed away in the back of a truck and welcome him to the group | MEL increased consistency of state 5. |
| 10  07:48 – 08:04 | The contortionist & the acrobat are seen performing | NON-MEL increased consistency of state 1. |
| 11  08:10 – 08:22 | The strongman & the flame thrower are seen performing | Nil |
| 12  09:01 – 09:47 | The trapeze artist is seen performing. The troupe are welcomed into a new town by the inhabitants | Nil |
| 13  09:57 – 10:03 | A father encourages his son that he can become ‘anything you want to be’ | Nil |
| 14  10:59 – 11:23 | The troupe perform for a small group, making the children laugh and bringing excitement about the coming show | Nil |
| 15  11:26 – 11:41 | Will and Mendez admire the circus performing | Nil |
| 16  12:35 – 12:43 | An old man down on his luck is invited to join the circus by Mendez | Nil |
| 17  13:13 – 13:25 | Mendez encourages Will that he can succeed despite his disabilities | Nil |
| 18  15:24 – 15:41 | Will summons the bravery to swim in the lake despite his fear of swimming | Nil |
| 19  16:51 – 16:59 | Will is cheered by the troupe after emerging from the lake | NON-MEL increased consistency of state 6. |
| 20  17:47 – 18:10 | Will performs in the circus, leaping from a height into a pool of water and amazing the crowd | Nil |
| 21  18:20 – 18:55 | Will inspires young people in the crowd who watch his performance | Nil |
| 22  19:07 – 19:30 | A young boy releases a butterfly that flies off into the distance | Nil |

**SUPPLEMENTARY TABLE 2**

Brain regions contributing to the positive valence map derived from neuroimaging meta-analysis. These regions were used for inference in the Hidden Markov Model. The centre of mass coordinates are reported in MNI152NLin6Asym space. Numbers in the first column correspond to 1 = left anterior insula, 2 = left posterior insula, 3 = right anterior insula, 4 = right posterior insula, 5 = bilateral superior anterior cingulate cortex, 6 = bilateral pregenual anterior cingulate cortex, 7 = bilateral subgenual anterior cingulate cortex, 8 = bilateral orbitofrontal cortex, 9 = right inferior frontal gyrus, 10 = left fusiform gyrus, 11 = right fusiform gyrus, 12 = right hippocampus, 13 = right amygdala, 14 = right thalamus, 15 = right nucleus accumbens, 16 = right globus pallidus, 17 = right putamen, 18 = right caudate, 19 = left hippocampus, 20 = left amygdala, 21 = left thalamus, 22 = left nucleus accumbens, 23 = left globus pallidus, 24 = left putamen, 25 = left caudate.

| Number | Region Name | Centre of Mass (x, y, z) | | |
| --- | --- | --- | --- | --- |
| 1 | L insula anterior | -35.81 | 15.61 | 0.10 |
| 2 | L insula posterior | -39.77 | -3.74 | 3.77 |
| 3 | R insula anterior | 40.80 | 15.15 | -2.97 |
| 4 | R insula posterior | 42.13 | -3.34 | 0.18 |
| 5 | sup ACC | 4.31 | 28.50 | 20.76 |
| 6 | pre ACC | 2.79 | 41.35 | 4.45 |
| 7 | sub ACC | 3.19 | 32.07 | -3.02 |
| 8 | OFC | 2.44 | 30.81 | -11.97 |
| 9 | R IFG | 35.80 | 21.04 | -16.87 |
| 10 | L Fusiform | -41.65 | -51.11 | -22.54 |
| 11 | R Fusiform | 37.40 | -51.17 | -19.61 |
| 12 | R HIP | 21.06 | -9.48 | -21.60 |
| 13 | R AMY | 23.79 | -4.37 | -18.96 |
| 14 | R THA | 8.27 | -12.73 | 3.84 |
| 15 | R NAc | 13.24 | 13.77 | -5.61 |
| 16 | R GP | 20.62 | -4.32 | -2.20 |
| 17 | R PUT | 27.27 | 0.07 | 0.20 |
| 18 | R CAU | 13.44 | 12.58 | 4.83 |
| 19 | L HIP | -19.88 | -11.08 | -19.43 |
| 20 | L AMY | -21.79 | -4.28 | -18.93 |
| 21 | L THA | -6.66 | -12.78 | 4.60 |
| 22 | L NAc | -10.14 | 13.40 | -5.73 |
| 23 | L GP | -17.10 | -3.39 | -2.27 |
| 24 | L PUT | -22.92 | 5.07 | -1.50 |
| 25 | L CAU | -10.32 | 9.93 | 5.91 |

**SUPPLEMENTARY TABLE 3**

Comparison of mean facial action unit activity during viewing of a stand-up comedian amongst participants with melancholic and non-melancholic depression

| Action Unit | Name | Mean Melancholic (sd) | Mean Non-Melancholic (sd) | $\boldsymbol{t}$-stat  \|t\| | Raw $\boldsymbol{p}$-value | FDR-corrected $\boldsymbol{p}$ | Hedges $\boldsymbol{g}$ |
| --- | --- | --- | --- | --- | --- | --- | --- |
| 01 | InnerBrowRaiser | 0.39 (0.23) | 0.47 (0.23) | 0.65 | 0.52 | 0.76 | 0.35 |
| 02 | OuterBrowRaiser | 0.43 (0.27) | 0.38 (0.24) | 0.40 | 0.69 | 0.85 | 0.20 |
| 04 | BrowLowerer | 0.65 (0.28) | 0.69 (0.30) | 0.25 | 0.80 | 0.85 | 0.14 |
| 05 | UpperLidRaiser | 0.22 (0.23) | 0.20 (0.20) | 0.34 | 0.74 | 0.85 | 0.09 |
| 06 | **CheekRaiser** | **0.49 (0.32)** | **0.91 (0.48)** | **3.02** | **0.0036** | **0.029 *** | **1.00** |
| 07 | **LidTightener** | **0.75 (0.44)** | **1.15 (0.51)** | **2.34** | **0.022** | **0.030 *** | **0.83** |
| 09 | **NoseWrinkler** | **0.59 (0.21)** | **0.87 (0.31)** | **2.11** | **0.039** | **0.040 *** | **1.03** |
| 10 | UpperLipRaiser | 0.53 (0.33) | 0.79 (0.40) | 1.82 | 0.073 | 0.17 | 0.70 |
| 12 | **LipCornerPuller** | **0.39 (0.40)** | **0.81 (0.56)** | **3.29** | **0.0016** | **0.026 *** | **0.84** |
| 14 | **Dimpler** | **0.45 (0.34)** | **0.82 (0.44)** | **2.88** | **0.0054** | **0.029 *** | **0.92** |
| 15 | LipCornerDepressor | 0.74 (0.30) | 0.73 (0.32) | 0.058 | 0.95 | 0.95 | 0.03 |
| 17 | ChinRaiser | 0.67 (0.29) | 0.58 (0.34) | 0.49 | 0.63 | 0.84 | 0.28 |
| 20 | LipStretcher | 0.58 (0.33) | 0.79 (0.35) | 1.60 | 0.12 | 0.24 | 0.61 |
| 23 | LipTightener | 0.41 (0.23) | 0.33 (0.22) | 1.13 | 0.26 | 0.46 | 0.36 |
| 25 | **LipParts** | **0.25 (0.28)** | **0.54 (0.49)** | **2.46** | **0.016** | **0.044 *** | **0.70** |
| 26 | JawDrop | 0.38 (0.28) | 0.47 (0.33) | 0.95 | 0.35 | 0.56 | 0.29 |

* = $p$ < 0.05 corrected for false discovery rate (FDR) using the method of Storey

**SUPPLEMENTARY TABLE 4**

Centre of mass coordinates of the two cerebellar clusters surviving cluster-level correction $p$_FWE_ < 0.05, where fMRI-inferred brain activity during positive scenes of an emotionally evocative movie correlated negatively (Figure 5) with mean facial activation unit intensity during viewing of a stand-up comedian. The coordinates are reported in MNI152NLin6Asym space.

| **Cerebellar Cluster** | **x** | **y** | **z** | **cluster-level p_FWE_** | **cluster extent** |
| --- | --- | --- | --- | --- | --- |
| Left Posterior Lobe | -31.56 | -57.27 | -33.90 | 0.002 | 1395 |
| Vermis | -2.42 | -61.09 | -6.58 | 0.022 | 728 |
